# Supplementary material for: Targeted single-cell genomics reveals novel host adaptation strategies of the symbiotic bacteria Endozoicomonas in Acropora tenuis coral
Source: Microbiome. 2022 Dec 12;10:220. doi: 10.1186/s40168-022-01395-9 (PMC9743535; doi:10.1186/s40168-022-01395-9)
Supplement: Supplementary file 2 — Additional file 1: Supplementary Figure 1. Droplet Digital PCR to quantify 16S rRNA gene copy number of bacteria. Supplementary Figure 2. Order level barplot and PCoA plot based on UniFrac distance of Endozoicomonas genus in four sampling points. Supplementary Figure 3. Average Nucleotide Identity (ANI) and Average Amino Acid Identity (AAI) of Endozoicomonas genomes. Supplementary Figure 4. Comparison of gene structures of Coral-like ephrin ligand genes. [file 40168_2022_1395_MOESM1_ESM.docx]

Supplementary files


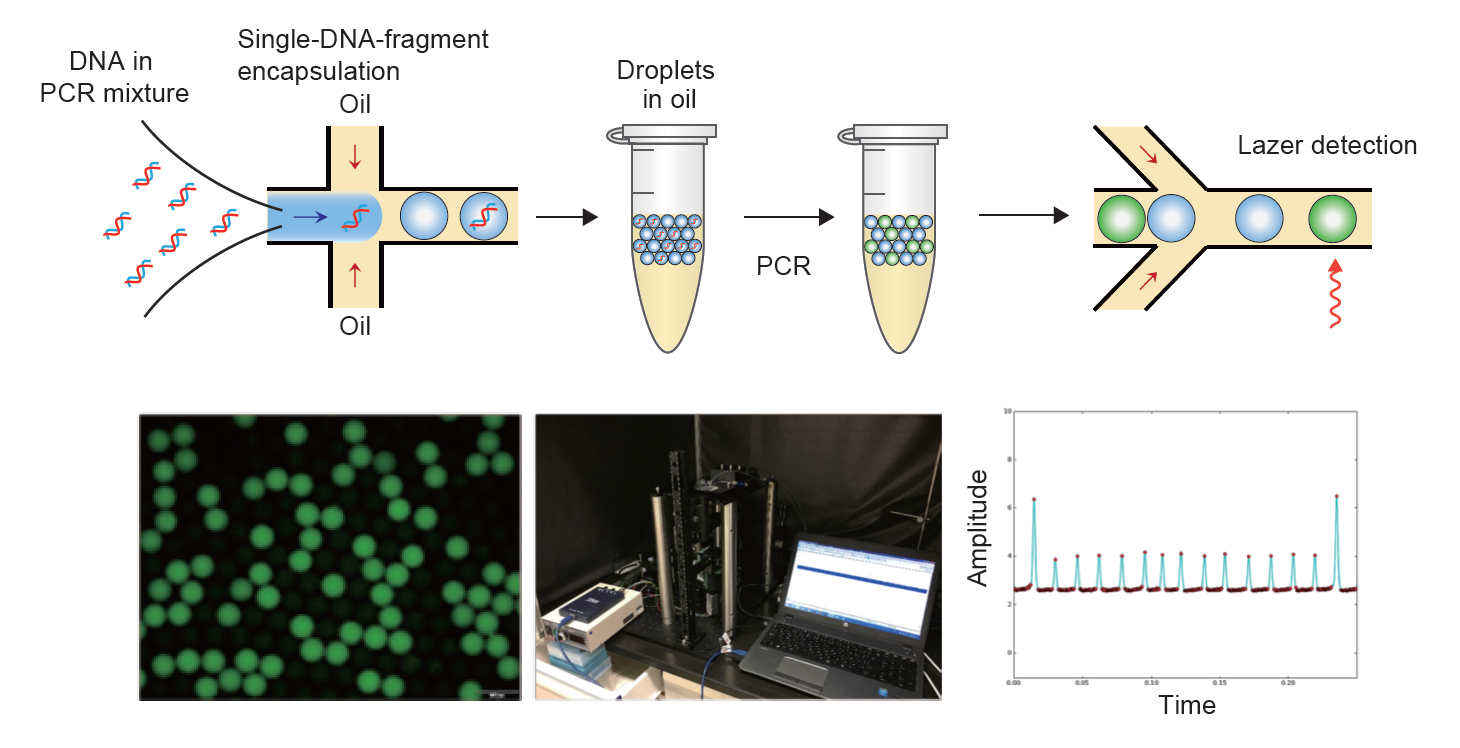


**Supplementary Figure 1. Droplet Digital PCR to quantify 16S rDNA copy number of bacteria**

The PCR mixture containing extracted DNA from coral branches was prepared and introduced into a microfluidic device for droplet generation. After thermal cycling, droplets were re-introduced into a microfluidic device for fluorescence detection. The number of total droplets and fluorescence-positive droplets were calculated with a custom designed optical system. The 16S rDNA copy number per 1 ng of DNA was calculated by the rate of fluorescence-positive droplets and the Poisson statistics.

##
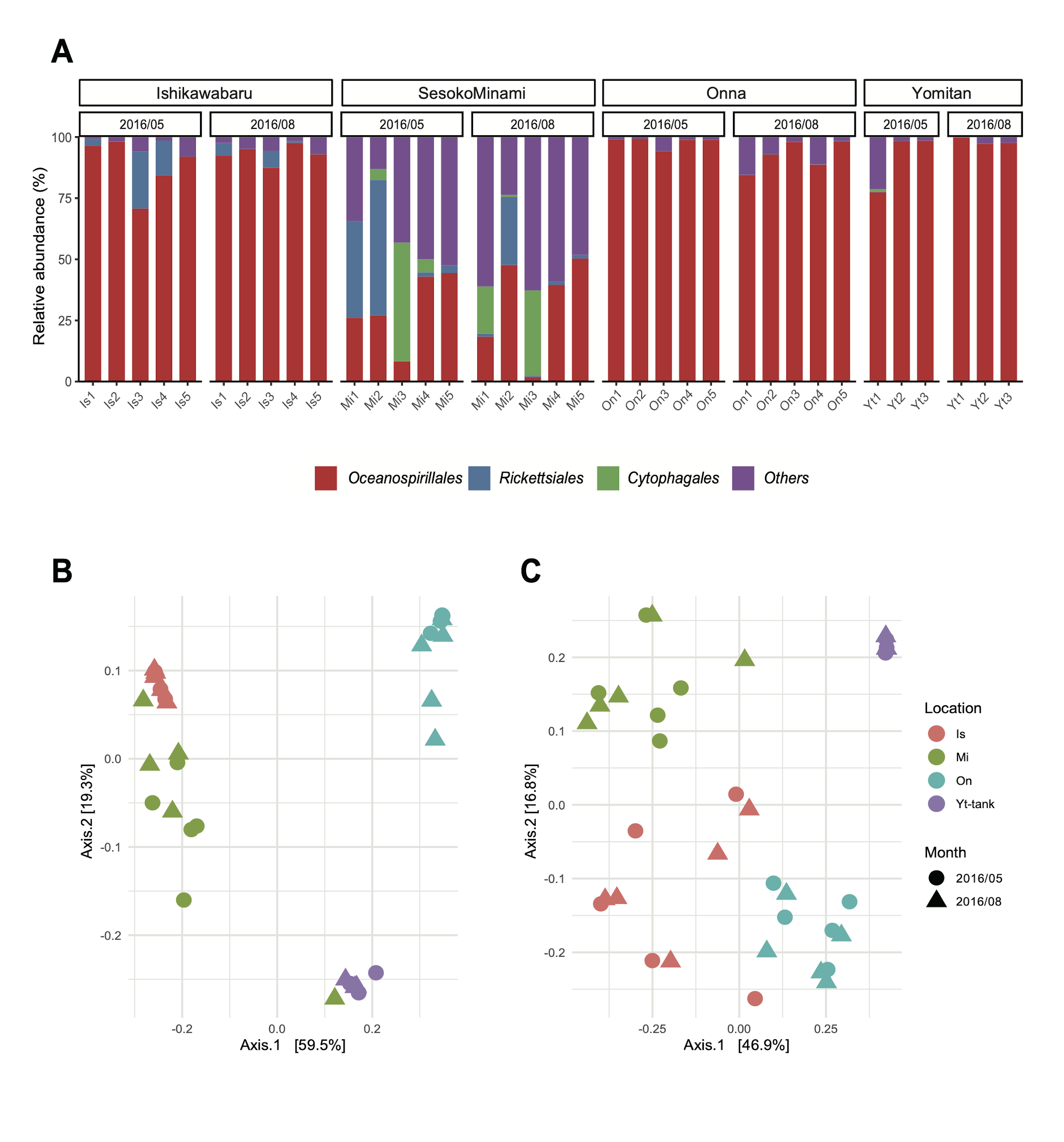


**Supplementary Figure 2. Order level barplot and PCoA plot based on UniFrac distance of Endozoicomonas genus in four sampling points**

A: Barplot of Order level, showing the major 3 orders.

B: PCoA plot of ASVs assigned to Endozoicomonas genus based on weighted UniFrac

C: PCoA plot of ASVs assigned to Endozoicomonas genus based on unweighted UniFrac


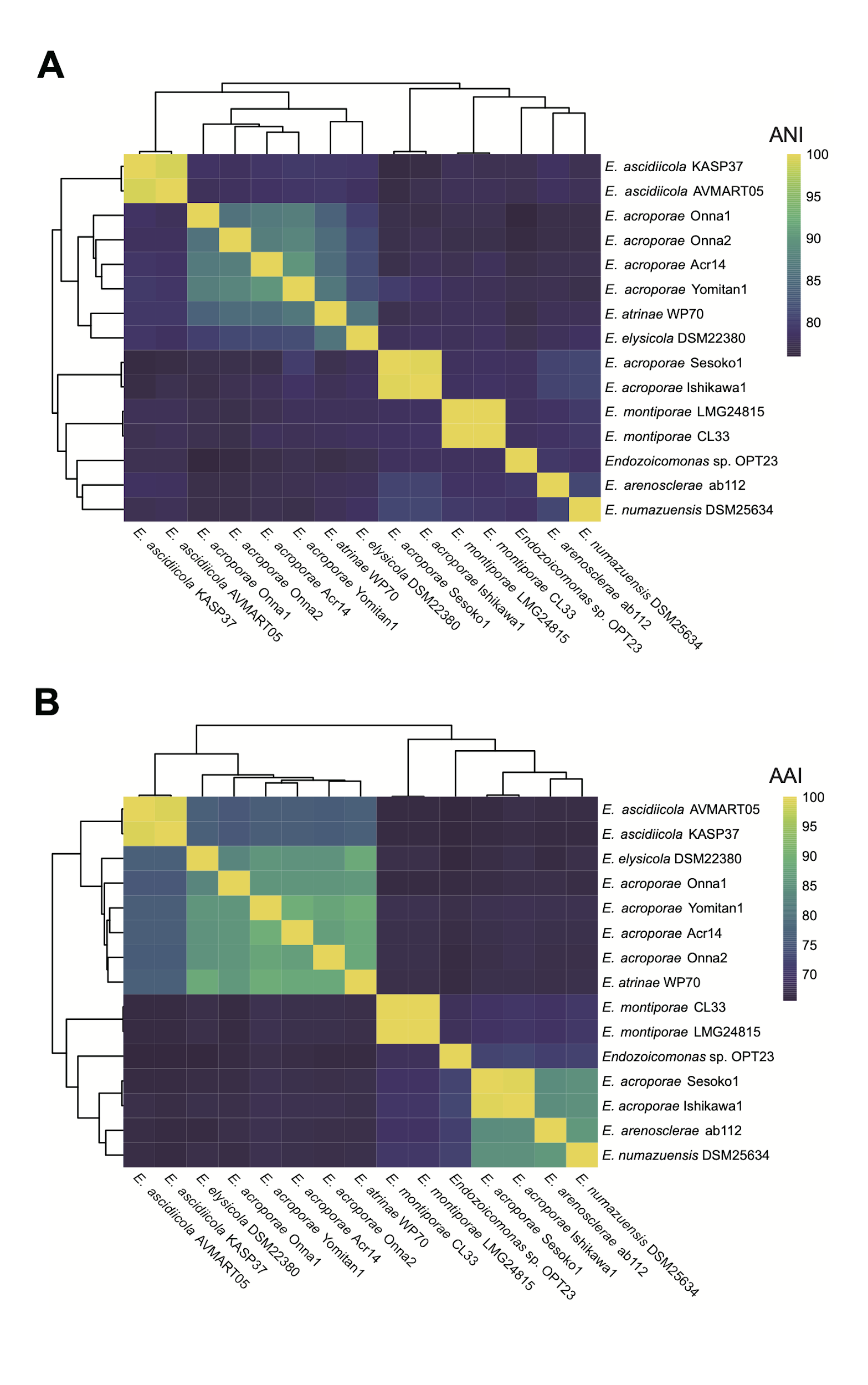


**Supplementary Figure 3. Average Nucleotide Identity (ANI) and Average Amino Acid Identity (AAI) of Endozoicomonas genomes**

A: Average Nucleotide Identity

B: Average Amino Acid Identity


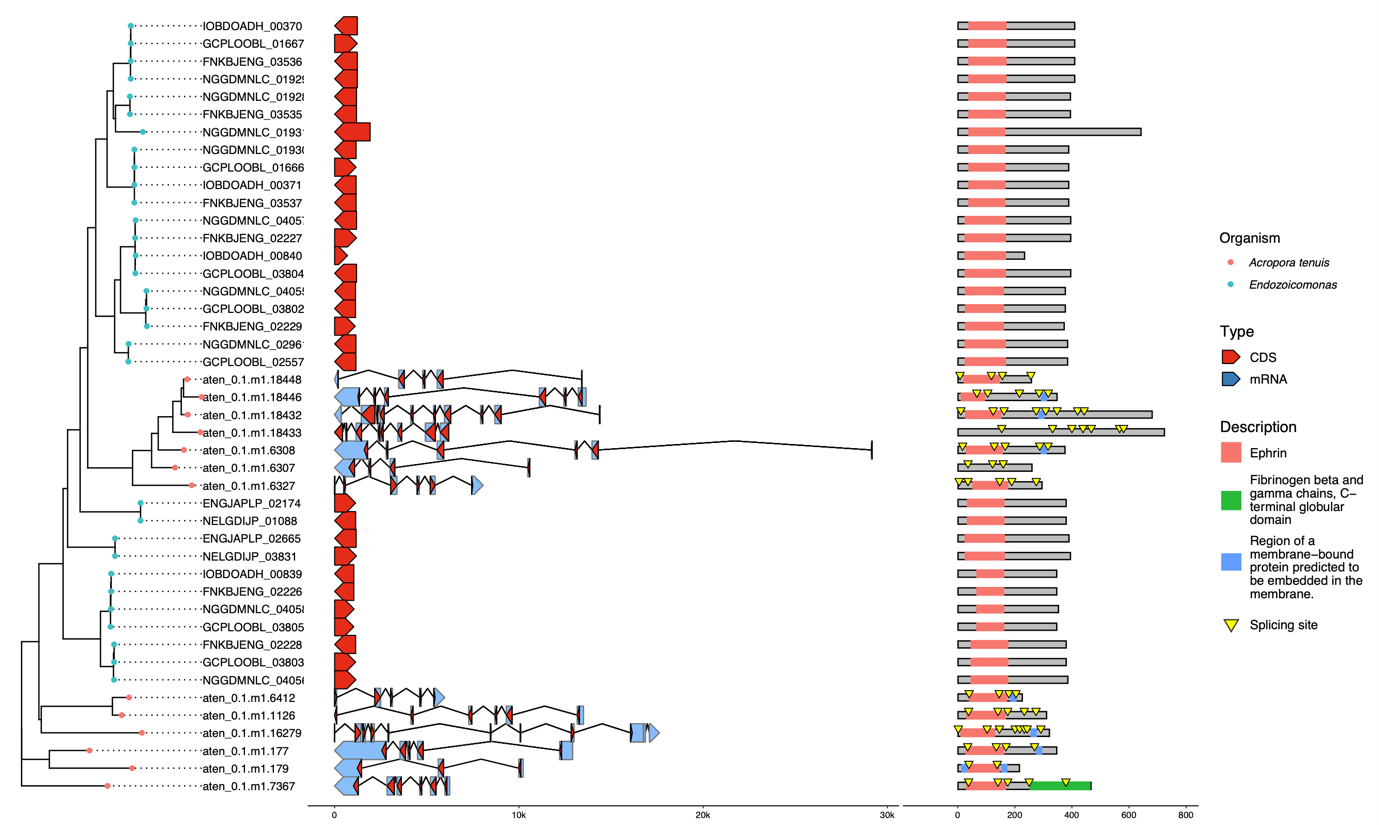


**Supplementary Figure 4. Comparison of gene structures of Coral-like ephrin genes |** The left column shows the phylogenetic tree was constructed by mafft and by IQ-TREE. In the middle column, the red arrows are the coding regions for amino acids. The blue arrows are untranslated eukaryotic mRNAs. The right column shows the domain structure of the protein, and the yellow triangles indicate splicing point.
